# Supplementary material for: Contrasting impacts of forests on cloud cover based on satellite observations
Source: Nat Commun. 2022 Feb 3;13:670. doi: 10.1038/s41467-022-28161-7 (PMC8813950; doi:10.1038/s41467-022-28161-7)
Supplement: Supplementary file 1 — Supplementary Information [file 41467_2022_28161_MOESM1_ESM.pdf]

Supplementary Information for Contrasting impacts of forests on cloud cover based on satellite observations

Ru Xu[1,2], Yan Li[1,2*], Adriaan J. Teuling[3], Lei Zhao[4,5], Dominick V. Spracklen[6], Luis Garcia-Carreras[7], Ronny Meier[8], Liang Chen[9], Youtong Zheng[10,11], Huiqing Lin[1,2], Bojie Fu[1,12]

1. State Key Laboratory of Earth Surface Processes and Resources Ecology, Beijing Normal University, Beijing 100875, China

2. Institute of Land Surface System and Sustainable Development, Faculty of Geographical Science, Beijing Normal University, Beijing 100875, China

3. Hydrology and Quantitative Water Management Group, Wageningen University and Research, Wageningen, The Netherlands.

4. Department of Civil & Environmental Engineering, University of Illinois at Urbana-Champaign, Urbana, IL 61801, USA.

5. National Center for Supercomputing Applications, University of Illinois at Urbana-Champaign, Urbana, IL 61801, USA.

6. School of Earth and Environment, University of Leeds, Leeds, LS2 9JT, UK

7. Centre for Atmospheric Science, Department of Earth and Environmental Sciences, University of Manchester, Manchester, M139PL, United Kingdom

8. Institute for Atmospheric and Climate Science, ETH Zurich, 8092 Zurich, Switzerland

9. Climate and Atmospheric Sciences Section, Illinois State Water Survey, Prairie Research Institute, University of Illinois at Urbana-Champaign, Champaign, IL 61820 USA

10. Earth System Science Interdisciplinary Center, University of Maryland, College Park, Maryland, 20742, USA.

11. Now at Program in Atmospheric and Oceanic Sciences/Geophysical Fluid Dynamics Lab, Princeton University, Princeton, NJ 08544 USA

12. State Key Laboratory of Urban and Regional Ecology, Research Center for Eco-Environmental Sciences, Chinese Academy of Sciences, Beijing, 100085, China

*Correspondence Yan Li: yanli.geo@gmail.com

Supplementary information includes Supplementary Figure 1 to 13 and Supplementary Table 1 to 3.

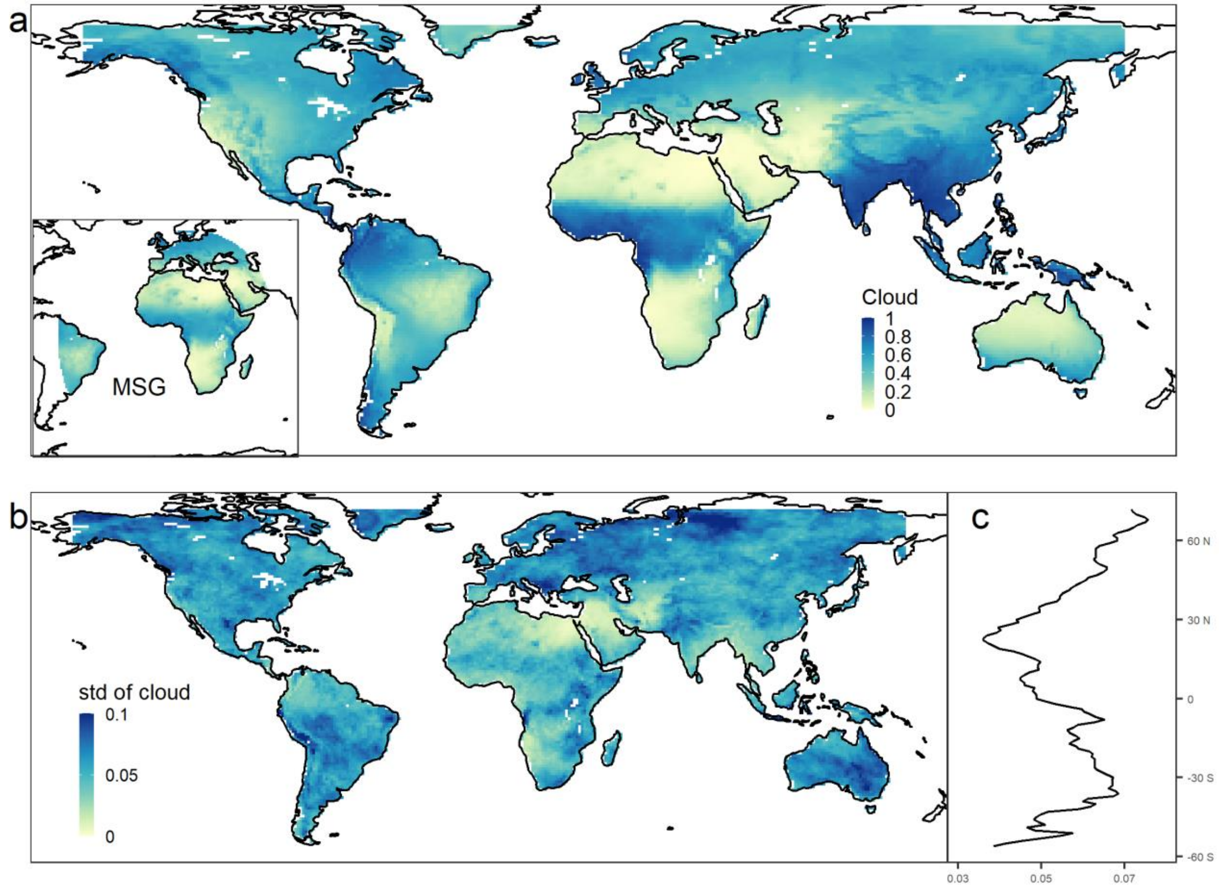

Supplementary Figure 1. (a) The spatial distribution of multi-year mean MODIS JJA cloud cover fractions at 0.05 ° from 2002 to 2018. (inset: MSG JJA cloud cover fractions from 2004 to 2013). (b) The standard deviation of the time series of MODIS JJA cloud cover fraction during 2002-2018 and (c) its latitudinal pattern.

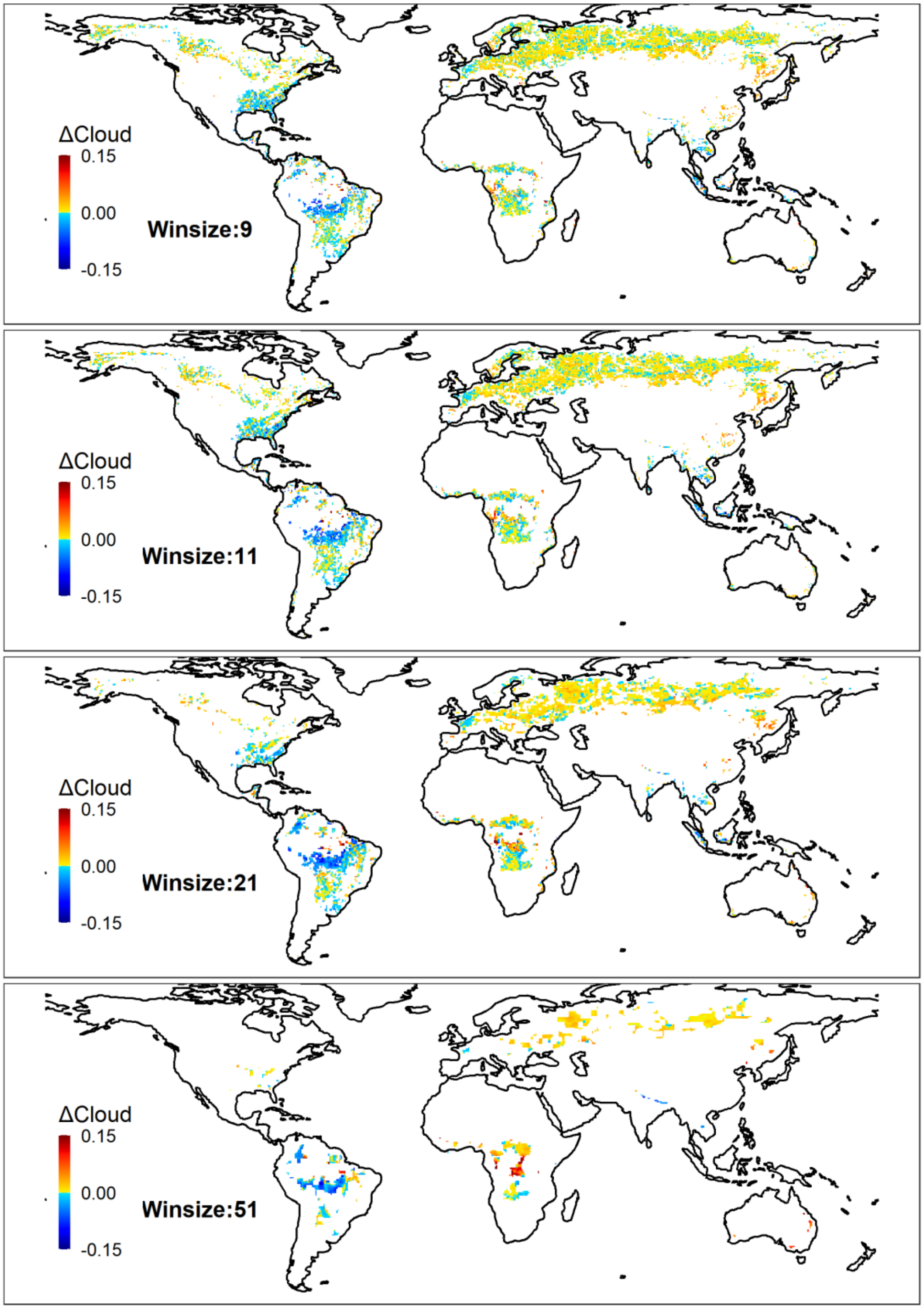

Supplementary Figure 2. The potential effects of forests on JJA cloud cover (ΔCloud) based on MODIS data at 0.05° resolution estimated using different window sizes: (a) 9×9 (0.45° × 0.45°), (b) 11×11 (0.55° × 0.55°), (c) 21×21 (1.05° × 1.05°), and (d) 51×51 (2.55° × 2.55°).

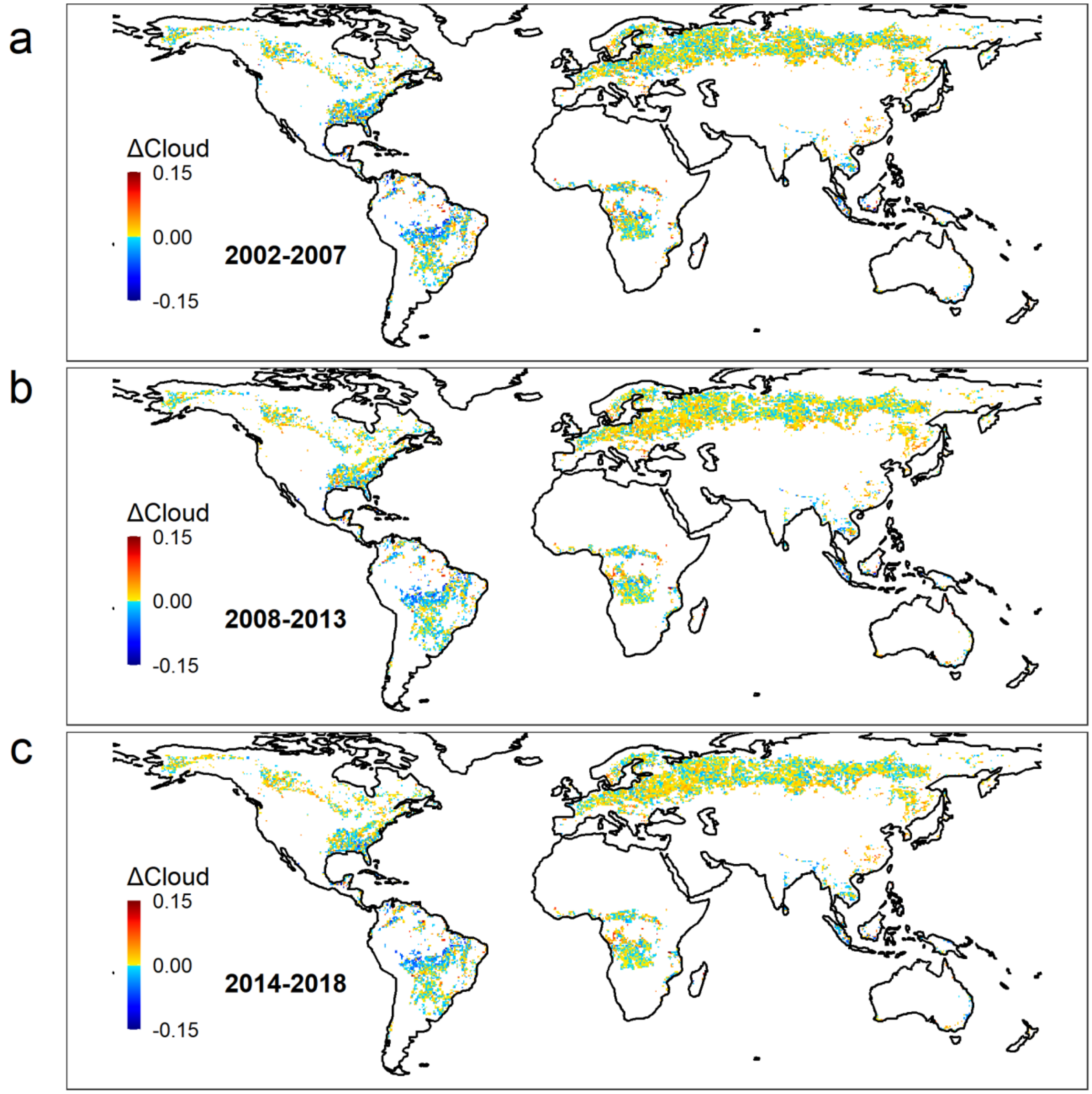

Supplementary Figure 3. The potential effects of forests on JJA cloud cover (ΔCloud) based on MODIS data estimated for different periods (a) 2002-2007 (b) 2008-2013 and (c) 2014-2018.

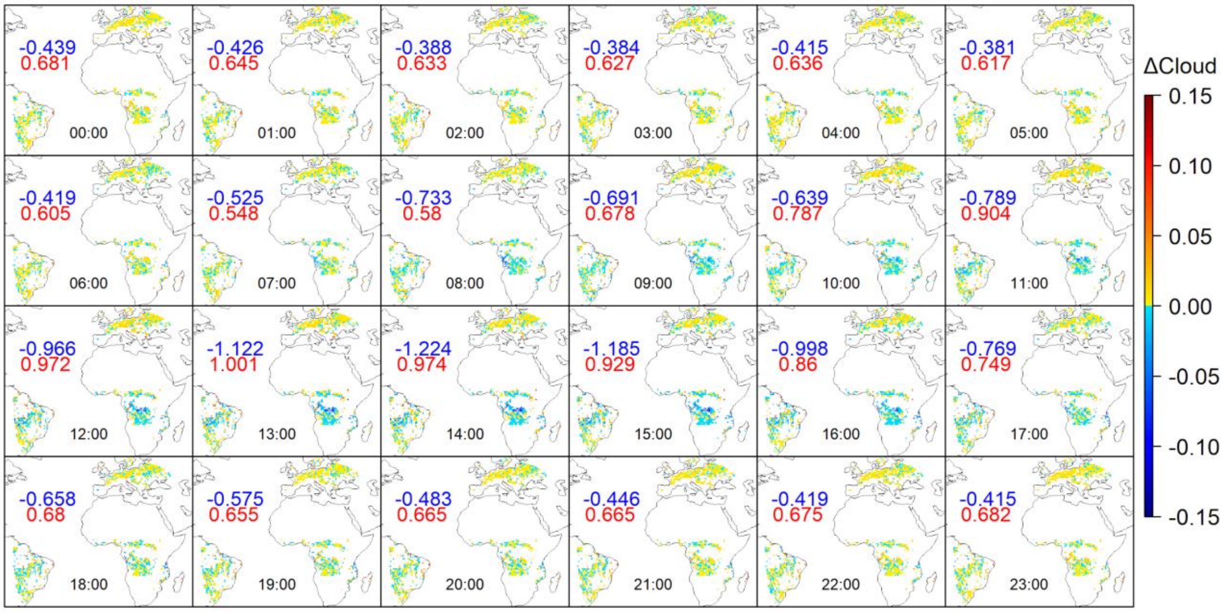

Supplementary Figure 4. Diurnal variations in the potential effects of forests on JJA cloud cover fraction (ΔCloud) based on MSG data. The red and blue texts show the averaged positive and negative ΔCloud over the domain, multiplied by 100 for display.

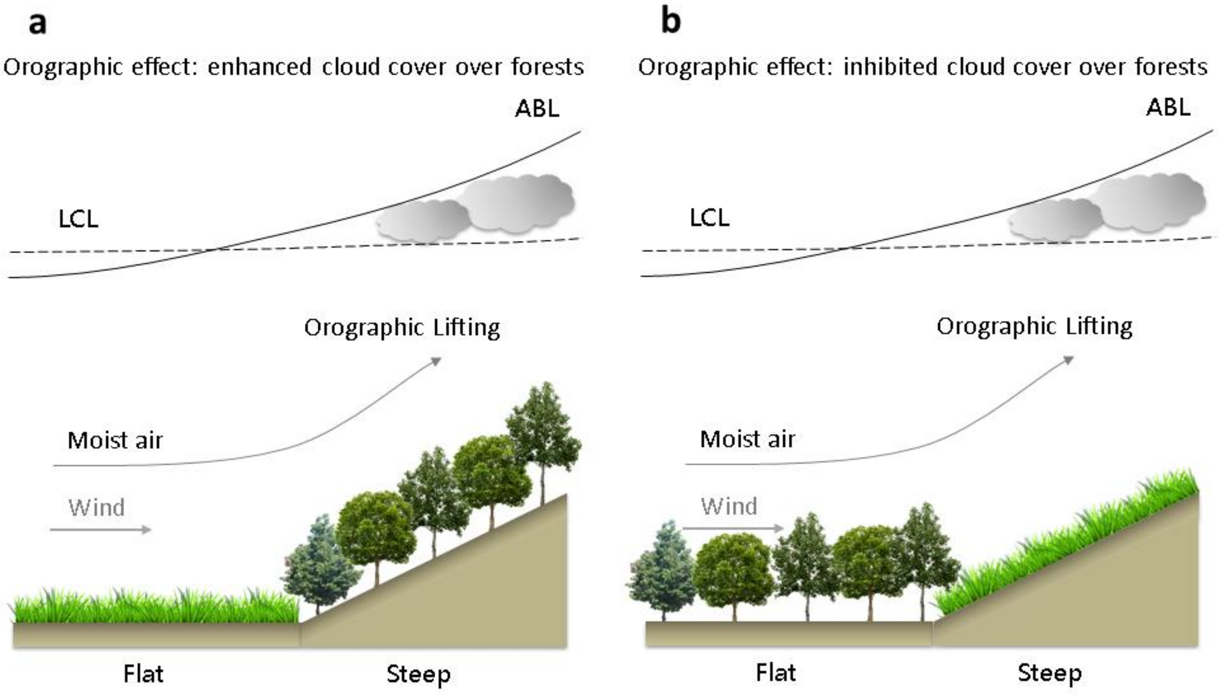

Supplementary Figure 5. Schematic of orographic clouds which could potentially confound the forest effects on cloud cover. (a) Orographic induced enhanced cloud cover and (b) inhibited cloud cover over forests.

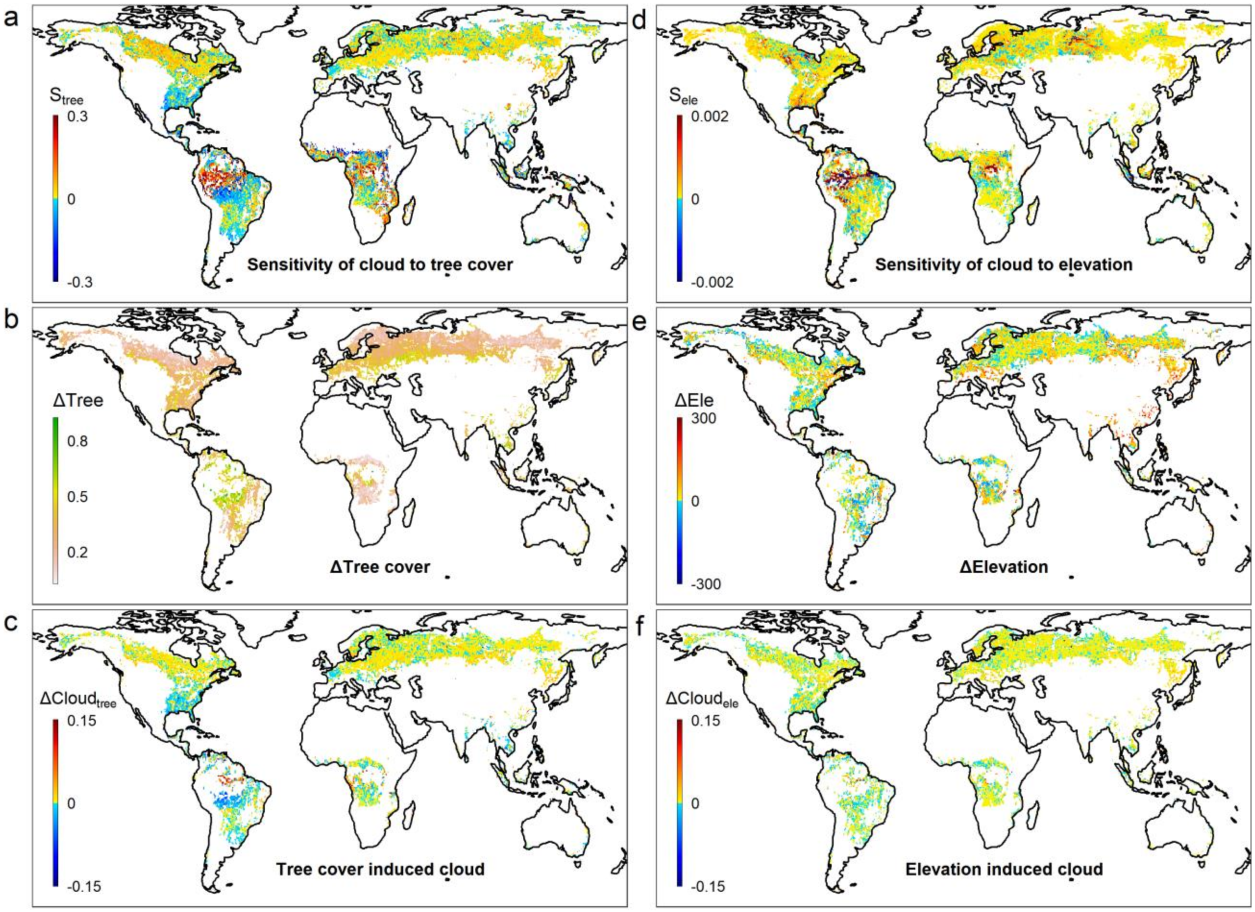

Supplementary Figure 6. Attribution of the potential cloud effects of forests in JJA (ΔCloud) to tree cover and elevation. (a,d) Sensitivities of cloud cover fraction to tree cover (S_{tree}, unit:fraction/fraction) and elevation (S_{ele}, unit:fraction/m) estimated using Eq. 4. (b,d) Differences between forests and non-forest in tree cover (ΔTree, unit:fraction) and elevation (ΔEle, unit:m). (c, f) Tree cover induced cloud differences estimated following Eq. 5 ($\Delta Cloud_{tree}$) and elevation induced cloud differences ($\Delta Cloud_{ele}$) estimated following Eq. 6.

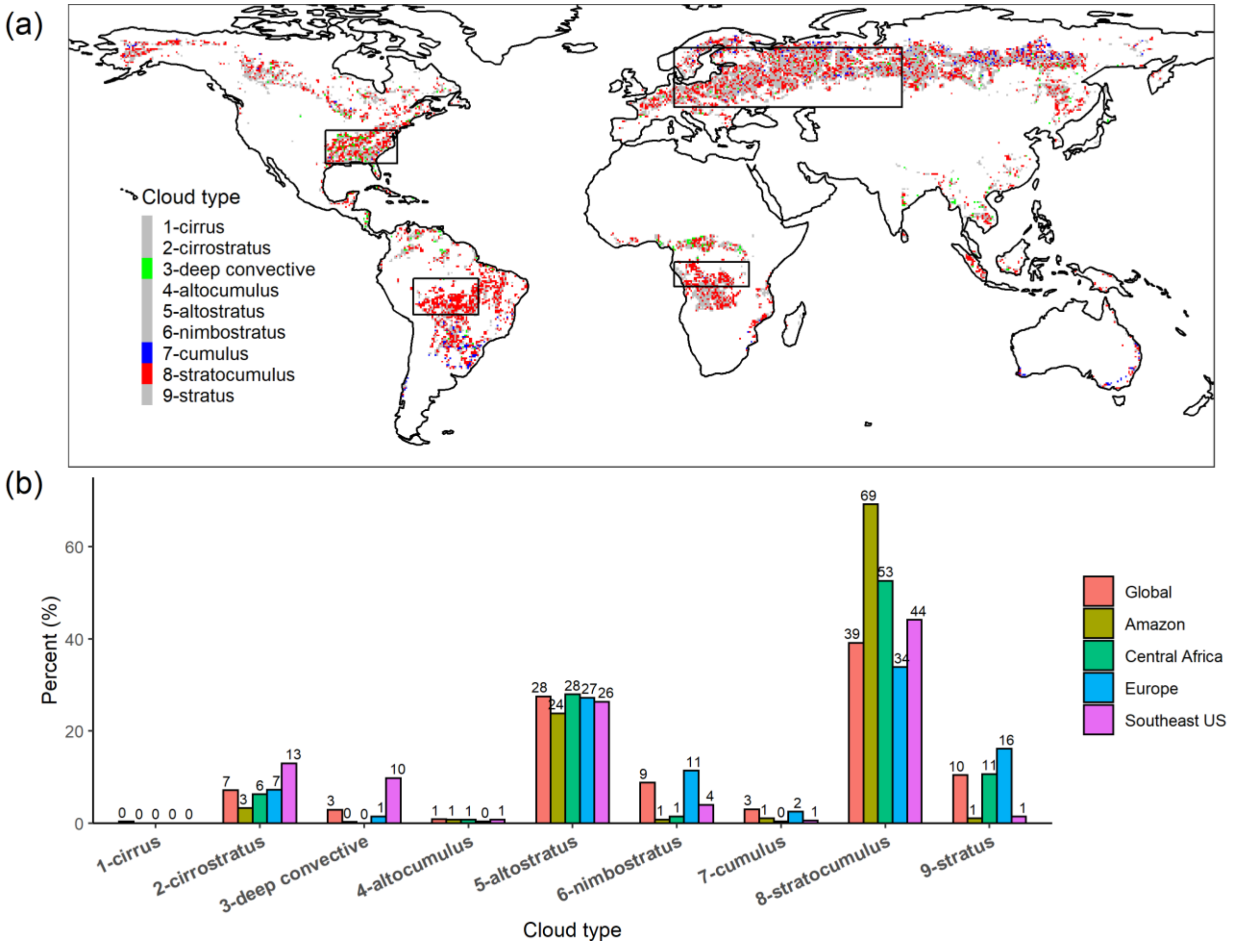

Supplementary Figure 7. (a) The dominant cloud type for the cloud effects of forests in JJA based on Sentinel-5P. Note that only the three convective cloud types are shown in color. (b) The percentage of each dominant cloud type globally and in four selected regions: Southeast US (97°W to 75°W, 30°N to 40°N), Amazon (70°W to 50°W, 16°S to 5°S), Central Africa (10°E to 33°E, 7.5°S to 0°) and Europe (10°E to 80°E, 47°N to 65°N). Note that the Europe box here is larger than that in Fig. 2.

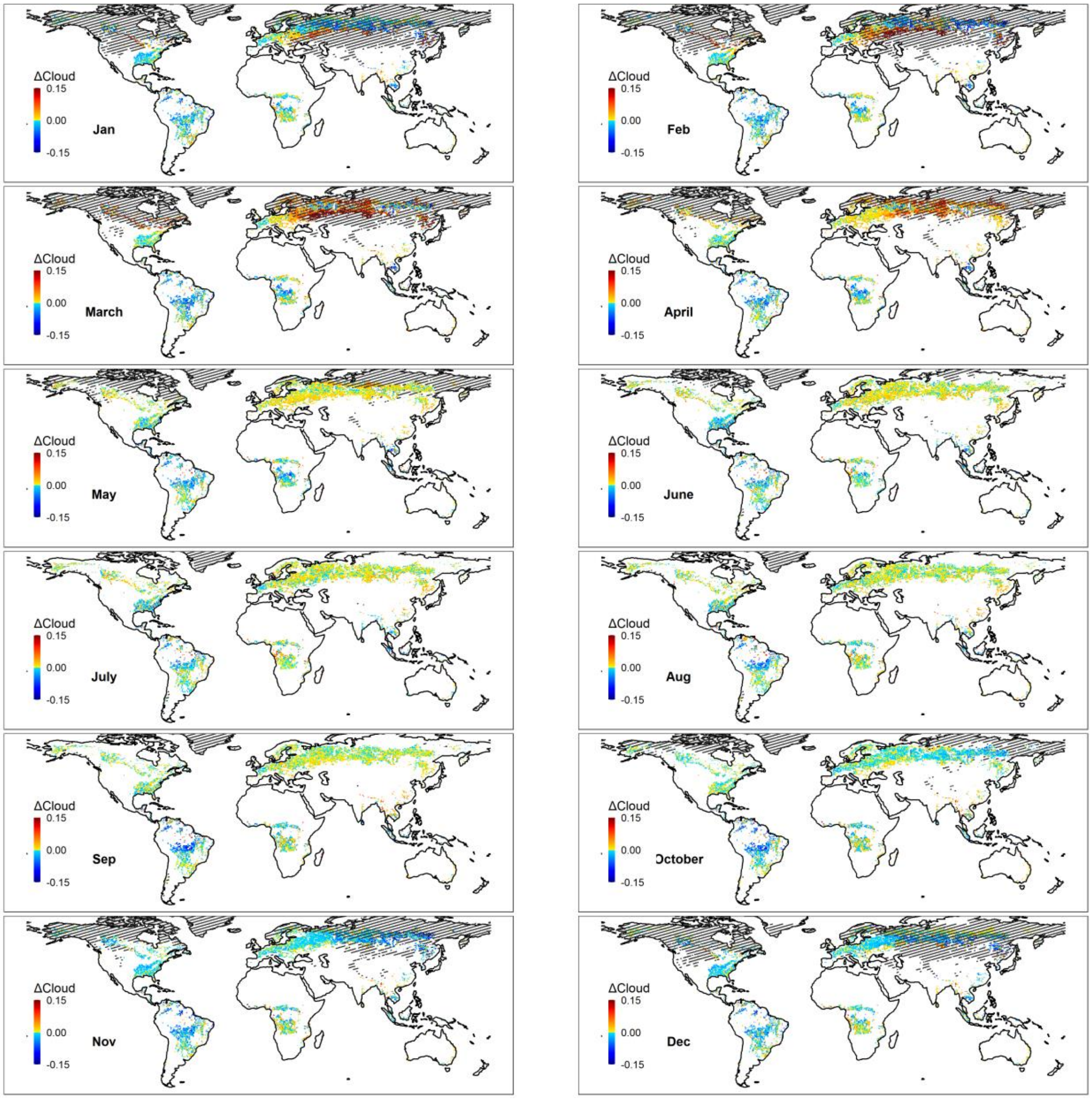

Supplementary Figure 8. Monthly variations in the potential effects of forests on cloud cover (ΔCloud) based on MODIS data. The presence of snow/ice is denoted as the dashed areas for each month.

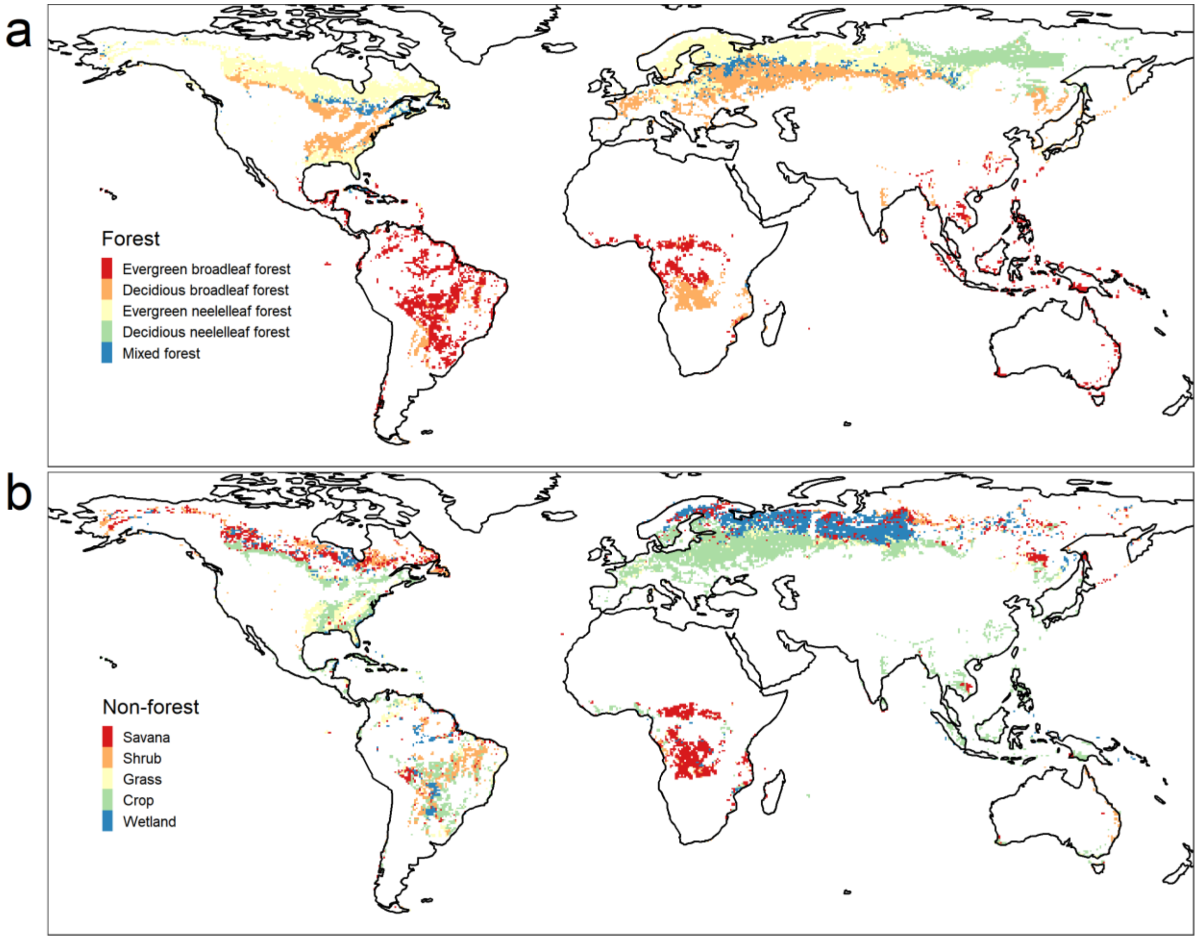

Supplementary Figure 9. Dominant land cover types for (a) forest and (b) non-forest pixels within the 9×9 moving window aggregated to 0.5° resolution. Land cover type information was from the ESA land cover data.

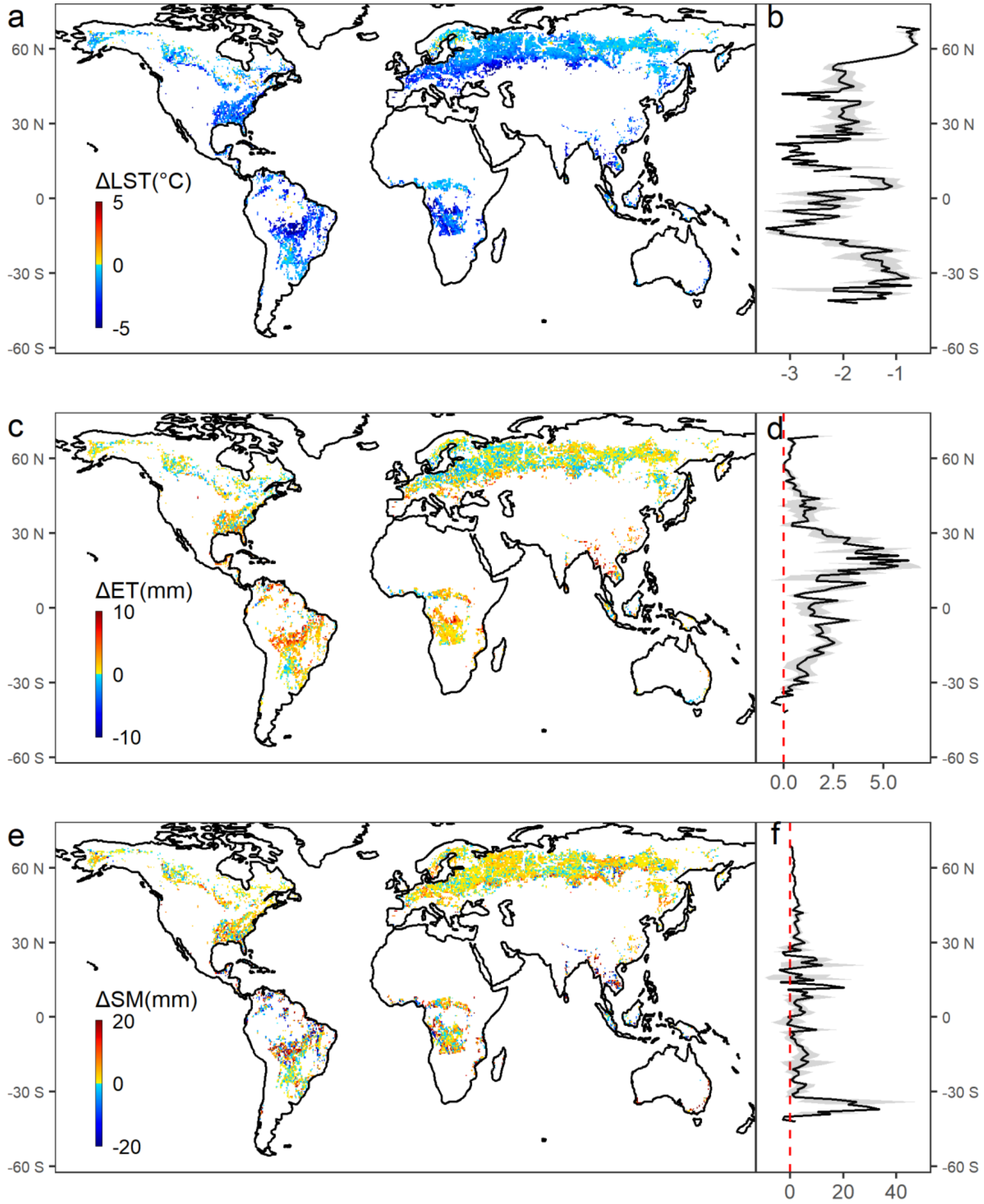

Supplementary Figure 10. The differences between forests and non-forest in LST (a), ET (c), and soil moisture (e) in JJA from 2002 to 2018 and their latitudinal patterns (b,d,f).

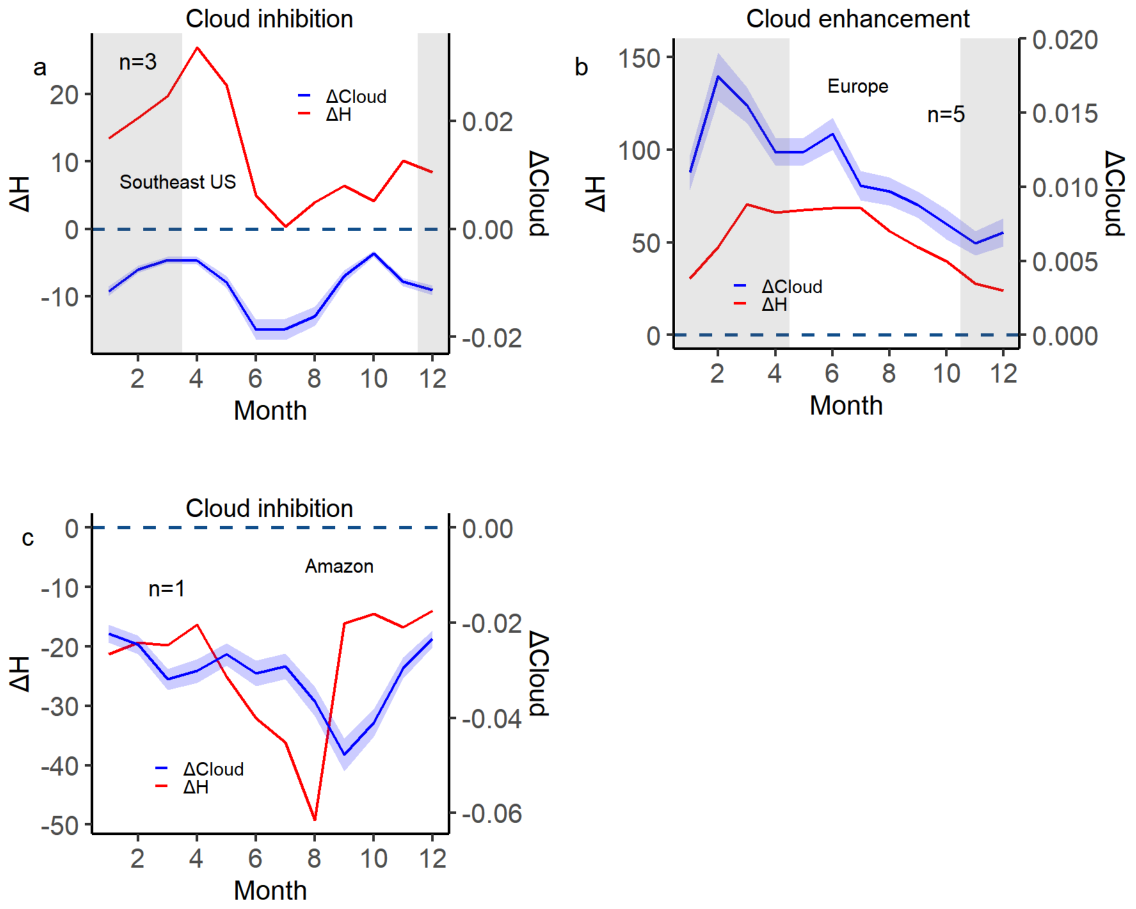

Supplementary Figure 11. The seasonal changes of ΔH from flux sites and MODIS ΔCloud for three selected regions: (a) Southeast US (97°W to 75°W, 30°N to 40°N), (b) Europe (10°E to 30°E, 47°N to 55°N), and (c) Amazon (70°W to 50°W, 16°S to 5°S). Months with snow cover are shown as shaded areas in Panels a-c. The n in each panel indicates the number of flux sites within each selected region (Table S1).

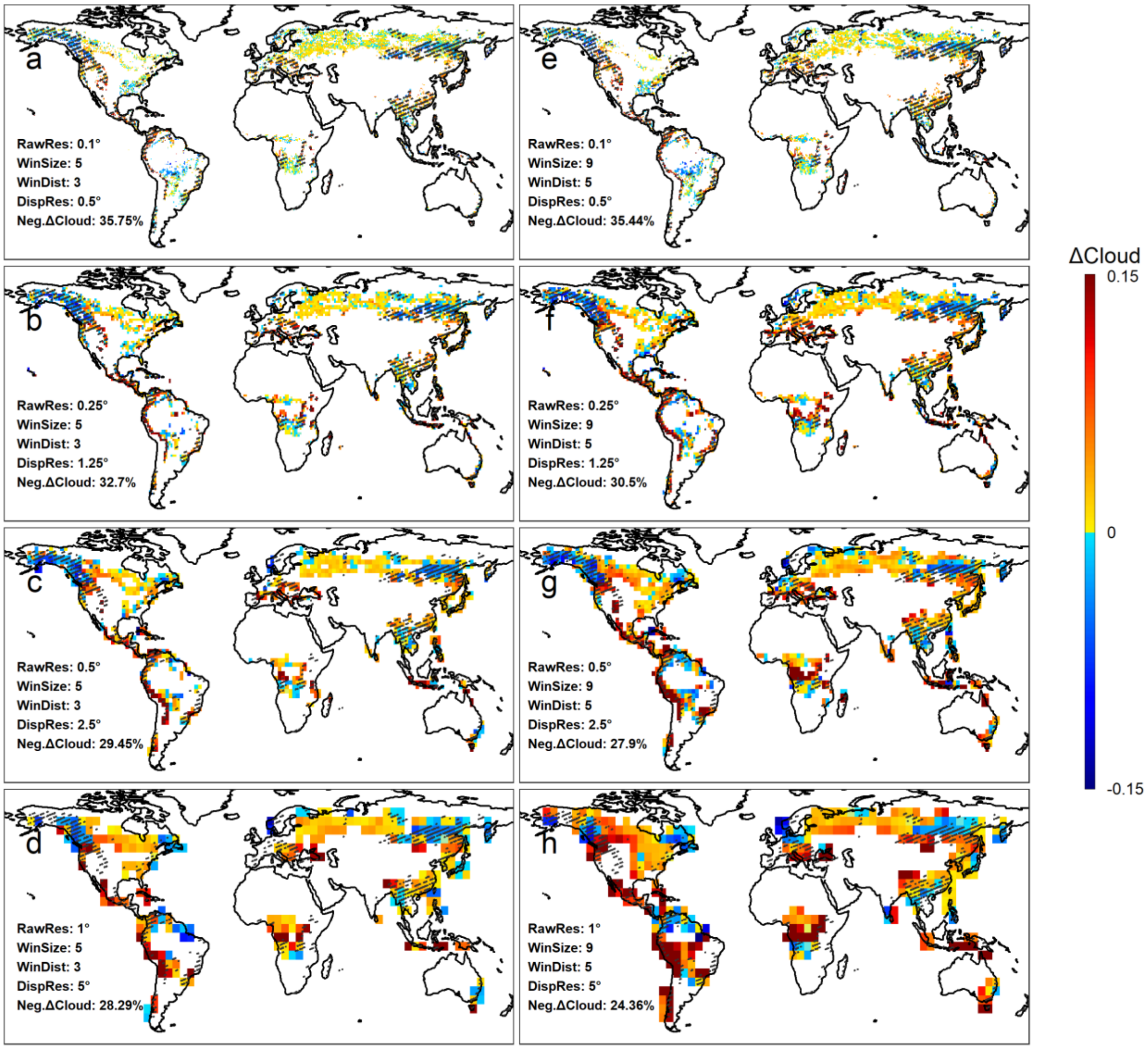

Supplementary Figure 12. The potential cloud effects of forests (ΔCloud) estimated using MODIS JJA cloud cover data resampled into different spatial resolutions at (a,e) 0.1°, (b,f) 0.25°, (c,g) 0.5°, and (d,h) 1°. Each column shows ΔCloud estimated using different parameter setups for window searching strategy (WinSize and WinDist, see Table S3). Dashed lines on the map show areas with complex topography (elevation sd. >100m). Note that the percentage of negative ΔCloud for different resolutions was calculated without excluding the areas with complex topography.

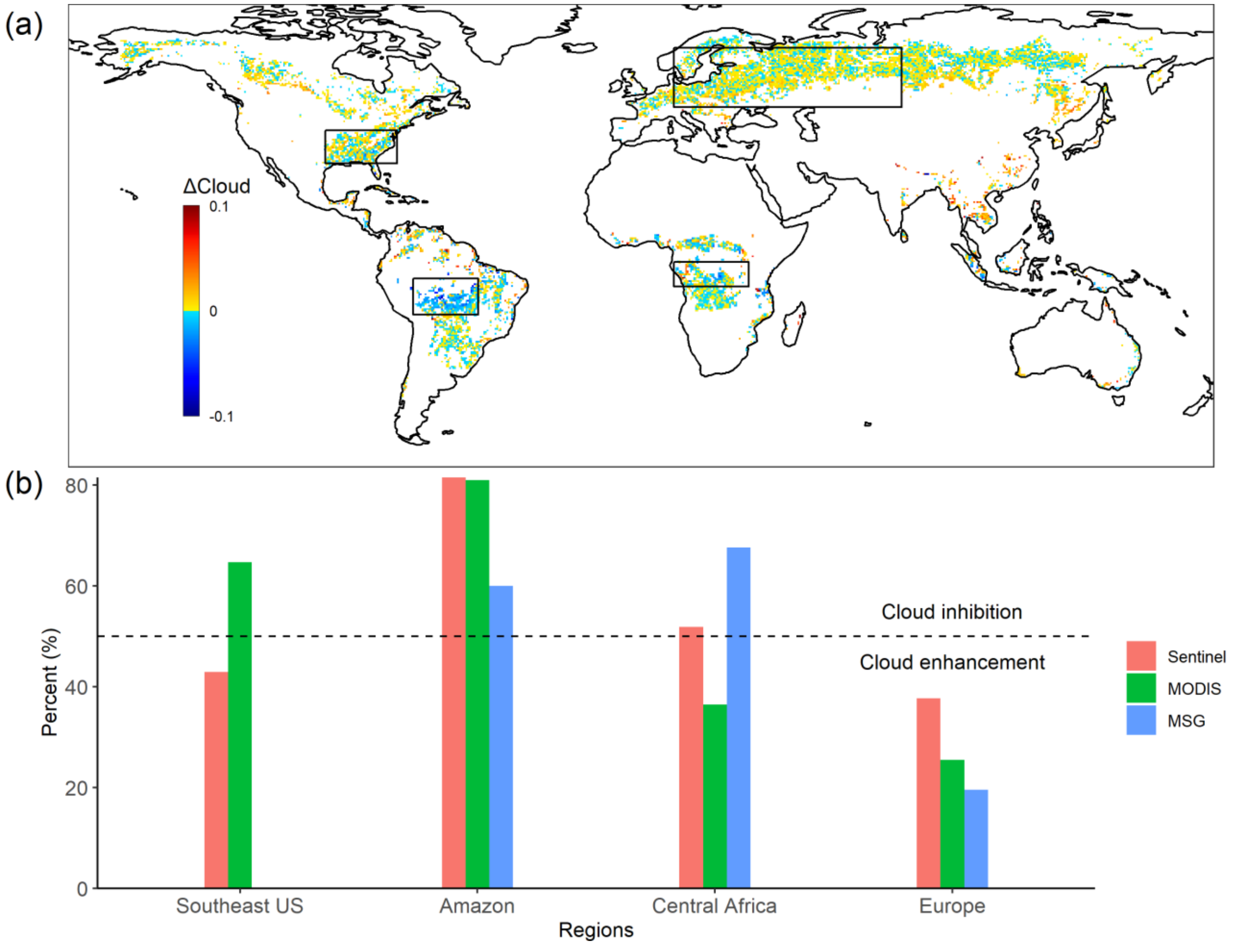

Supplementary Figure 13. (a) The potential effect of forests on cloud cover (ΔCloud) based on Sentinel-5P data and (b) the percentage of negative ΔCloud in four selected regions from Sentinel-5P, MODIS, and MSG cloud cover data. The four black rectangles in panel (a) denote four hotspots regions, Southeast US (97°W to 75°W, 30°N to 40°N), Amazon (70°W to 50°W, 16°S to 5°S), Central Africa (10°E to 33°E, 7.5°S to 0°) and Europe (10°E to 80°E, 47°N to 65°N). Note that the Europe box here is larger than that in Fig. 2. The dashed black horizontal line in panel (b) represents the 50% percent line, with value >50% indicating more cloud inhibition and <50% indicating more cloud enhancement of forests.

Supplementary Table 1. Paired forest and non-forest flux sites used in this study. The rightmost column denotes flux sites used in Supplementary Figure 11.

Pair number	Non-forest site	Forest site	Non-forest site latitude	Non-forest site longitude	Forest site latitude	Forest site longitude	Non-forest land cover	Region
1	FR-Gri	FR-Fon	48.8442	1.9519	48.4764	2.7801	Cropland	
2	NL-Hor	NL-Loo	52.2404	5.0713	52.1666	5.7436	Grassland	
3	DE-Gri	DE-Tha	50.9495	13.5125	50.9636	13.5669	Grassland	Europe
4	DE-Kli	DE-Tha	50.8929	13.5225	50.9636	13.5669	Grassland	Europe
5	CA-NS6	CA-NS2	55.9167	-98.9644	55.9058	-98.5247	Open Shrubland	
6	CA-NS6	CA-NS5	55.9167	-98.9644	55.8631	-98.485	Open Shrubland	
7	CA-NS6	CA-NS1	55.9167	-98.9644	55.8792	-98.4839	Open Shrubland	
8	CA-NS6	CA-NS3	55.9167	-98.9644	55.9117	-98.3822	Open Shrubland	
9	CA-SF3	CA-SF1	54.0916	-106.005	54.485	-105.818	Open Shrubland	
10	CA-SF3	CA-SF2	54.0916	-106.005	54.2539	-105.878	Open Shrubland	
11	BE-Lon	BE-Vie	50.5515	4.7461	50.305	5.998	Cropland	
12	US-Wi6	US-Wi0	46.6249	-91.2982	46.6188	-91.0814	Open Shrubland	
13	US-Wi6	US-Wi3	46.6249	-91.2982	46.6347	-91.0987	Open Shrubland	
14	US-Wi6	US-Wi4	46.6249	-91.2982	46.7393	-91.1663	Open Shrubland	
15	AU-Rig	AU-Whr	-36.6499	145.5759	-36.6732	145.0294	Grassland	
16	IT-CA2	IT-CA1	42.3772	12.026	42.3772	12.026	Cropland	
17	IT-CA2	IT-CA3	42.3772	12.026	42.38	12.0222	Cropland	
18	DE-RuS	BE-Vie	50.8659	6.4472	50.3051	5.9981	Cropland	
19	CZ-BK2	CZ-BK1	49.4944	18.5429	49.5021	18.5369	Grassland	Europe
20	US-Var	US-Blo	38.4133	-120.951	38.8953	-120.633	Grassland	
21	IT-CA2	IT-Ro2	42.3772	12.026	42.3903	11.9209	Cropland	
22	AT-Neu	IT-Ren	47.1167	11.3175	46.5869	11.4337	Grassland	
23	DE-Kli	DE-Obe	50.8929	13.5225	50.7836	13.7196	Cropland	Europe
24	DE-Gri	DE-Obe	50.9495	13.5125	50.7836	13.7196	Grassland	Europe
25	US-Dk1	US-Dk2	35.9712	-79.0934	35.9736	-79.1004	Grassland	Southeast US
26	US-Dk1	US-Dk3	35.9712	-79.0934	35.9782	-79.0942	Grassland	Southeast US
27	US-NC1	US-NC2	35.8118	-76.7119	35.803	-76.6685	Open Shrubland	Southeast US

28	US-Fwf	US-Fmf	35.4435	-111.772	35.1426	-111.727	Grassland	
29	STM_K77	STM_K83	-3.0202	-54.8885	-3.017	-54.9707	Cropland	
30	RON_FNS	RON_RJA	-10.7618	-62.3572	-10.078	-61.9331	Pasture	Amazon

Supplementary Table 2. Parameter sets of window searching strategy for cloud cover data with different spatial resolutions. Parameters include raw data resolution (RawRes), window size (WinSize), window distance (WinDist), resolution for display (DisRes), and percent of negative ΔCloud. There are two parameter combinations for each resolution. The percentage of negative ΔCloud for different resolutions was calculated based on Fig. S12, without excluding areas with complex topography

RawRes	WinSize	WinDist	DispRes	Negative ΔCloud percent (%)
0.05°	9	5	0.5°	36.57
0.1°	9	5	0.5°	35.44
	5	3		35.75
0.25°	9	5	1.25°	30.50
	5	3		32.70
0.5°	9	5	2.5°	27.90
	5	3		29.45
1°	9	5	5°	24.36
	5	3		28.29

Supplementary Table 3. Lookup table of converting CLM land classification scheme to IGBP scheme

CLM scheme*	IGBP scheme
4,5	broadleaf evergreen forest
6,7,8	broadleaf deciduous forest
1,2	needleleaf evergreen forest
3	needleleaf deciduous forest
1-8	mixed forest
1-11	savannas
9-11	shrubland
12-14	grass
15	crop

*CLM land classification scheme: 1 needleleaf evergreen temperate tree, 2 needleleaf evergreen boreal tree, 3 needleleaf deciduous boreal tree, 4 broadleaf evergreen tropical tree, 5 broadleaf evergreen temperate tree, 6 broadleaf deciduous tropical tree, 7 broadleaf deciduous temperate tree, 8 broadleaf deciduous boreal tree, 9 broadleaf evergreen temperate shrub, 10 broadleaf deciduous temperate shrub, 11 broadleaf deciduous boreal shrub, 12 arctic c3 grass, 13 cool c3 grass, 14 warm c4 grass, 15 crop
